# Limitations of Mind

**Published:** 1884-04

**Authors:** 


### LIMITATIONS OF MIND.

NOW, it may be asked, "Does this line of reasoning prove there is no God?" asks N. J. Gates. Not at all; it simply proves that the finite mind is utterly impotent to apprehend God. It proves that we do not and cannot comprehend primary causations; that our perceptive faculties are so limited by the very nature of their constitution that they cannot apprehend the primary nature of the simplest natural law; and if we cannot comprehend the nature of the force called gravity, or heat, as a mode of motion, except as physical facts, how can we have any rational conception of any of those matchless qualities of Mind That produce these laws? If the rude savage, after examining for the first time, a complicated piece of machinery, can form no judgment, can form no just conception of the forces that impel it or even of the purpose it serves, how much less can he understand the peculiar qualities of mind that invented and produced it? If, by dint of the deepest research, we cannot analyze the subtle law that connects the molecular movement of the brain with thought, how can we analyze the thoughts of an Infinite Mind of Which this law was but a thought? Is it not piain that, in attempting this, we attempt the impossible?

A striking article in the April *Century*, by Walter B. Hill, on "Uncle Tom without a Cabin," opens with the following reminesces of slavery days: "In the last year of his life, General 'Light Horse Harry' Lee made a visit to Dungeness, the residence of General Nathaniel Green, on Cumberland Island, Georgia. While there he was attacked with a sickness which in the end proved fatal. His nurse was an old negro woman, the 'momma' of the household. One day, in a paroxysm of nervous pain, he became enraged at her officious benevolence, and threw a slipper at the old woman's head. There was a skillful dodge of the red bandanna, and then she deliberately picked up the slipper and hurled it back at him, with the words, 'Dah, now! I aint gwine to let no white chile sass me; *I* aint. This incident illustrates the position of the 'momma' in a Southern family in the olden time. She had rocked the cradle of her young master, and as he grew to manhood he was still her 'chile.'

Perhaps you smoke and chew! What's the use of paying out $100 a year to insure bad breath, headache, red eyes, decayed teeth, and nervous debility, when you can secure a broken leg, which is far nicer, by a tumble down stairs?

